# Functional Nanomaterial-Based Electrochemical Biosensors Enable Sensitive Detection of Disease-Related Small-Molecule Biomarkers for Diagnostics

**DOI:** 10.3390/ph19020223

**Published:** 2026-01-27

**Authors:** Tongtong Xun, Jie Zhang, Xiaojuan Zhang, Min Wu, Yueyan Huang, Huanmi Jiang, Xiaoqin Zhang, Baoyue Ding

**Affiliations:** 1 College of Life Science and Medicine, Zhejiang Sci-Tech University, Hangzhou 310018, China; xuntongtong111@163.com; 2 Jiaxing Key Laboratory for Photonanomedicine and Experimental Therapeutics, Department of Pharmaceutics, College of Medicine, Jiaxing University, Jiaxing 314001, China; zhangjiepharm@zjxu.edu.cn (J.Z.); xjzhang@zjxu.edu.cn (X.Z.); minwu220@zjxu.edu.cn (M.W.); hyylinda@163.com (Y.H.); jianghuanmi233@126.com (H.J.)

**Keywords:** electrochemical sensors, biomolecule detection, glucose, glutathione, dopamine, uric acid, lactate, cholesterol

## Abstract

Biomolecules play pivotal roles in cellular signaling, metabolic regulation and the maintenance of physiological homeostasis in the human body, and their dysregulation is closely associated with the onset and progression of various human diseases. Consequently, the development of highly sensitive, selective, and stable detection platforms for these molecules is of significant value for drug discovery, pharmaceutical quality control, pharmacodynamic studies, and personalized medicine. In recent years, electrochemical biosensors, particularly those integrated with functional nanomaterials and biorecognition elements, have emerged as powerful analytical platforms in pharmaceutics and biomedical analysis, owing to their high sensitivity, exquisite selectivity, rapid response, simple operation, low cost and suitability for real-time or in situ monitoring in complex biological systems. This review summarizes recent progress in the electrochemical detection of representative biomolecules, including dopamine, glucose, uric acid, hydrogen peroxide, lactate, glutathione and cholesterol. By systematically summarizing and analyzing existing sensing strategies and nanomaterial-based sensor designs, this review aims to provide new insights for the interdisciplinary integration of pharmaceutics, nanomedicine, and electrochemical biosensing, and to promote the translational application of these sensing technologies in drug analysis, quality assessment, and clinical diagnostics.

## 1. Introduction

Small biological molecules, as the fundamental substrates underpinning life processes, are indispensable for maintaining normal physiological functions in the human body. These molecules, including dopamine, glucose, hydrogen peroxide, among others, participate in neural functions, metabolic processes, and various physiological activities, thereby playing a critical role in the proper functioning of the organism [1,2,3]. In the complex physiological platforms of the human body, various biological small molecules such as metabolites, fatty acids, neurotransmitters, amino acids, and hormones play crucial roles. These molecules are constantly produced, diffused, and consumed under physiological conditions, thereby maintaining the normal functioning of the human body. However, external environmental stimuli or pathological factors can disrupt the physiological dynamic balance, causing irregular changes in intracellular concentrations of small biological molecules. This can trigger metabolic disorders, signal transduction dysregulation, and other issues that affect human health, becoming an important molecular basis for the occurrence and development of various diseases [4].

From a biomedical and pharmaceutical perspective, many of these small biomolecules also serve as important disease biomarkers and therapeutic targets, and their dynamic fluctuations are closely related to drug efficacy, adverse drug reactions, and individualized treatment outcomes. Therefore, sensitive and accurate monitoring of key small biomolecules is of great significance for early disease diagnosis, therapeutic evaluation, and the optimization of drug therapy regimens. Given the crucial roles of biological small molecules in both physiological regulation and pharmacotherapy, the engineering of rapid, highly specific, sensitive and robust detection platforms has emerged as a paramount endeavor of current research. In recent years, electrochemical sensors have demonstrated significant potential in this field due to their unique advantages. Compared to traditional analytical techniques such as chromatography, spectroscopy, and mass spectrometry, electrochemical sensors offer multiple benefits in practical applications, including high sensitivity, exquisite selectivity, short response times, ease of operation, low cost, and the ability to perform real-time monitoring. These advantages have led to their rapid development in areas such as clinical diagnosis, environmental monitoring, food safety, and life sciences [5,6,7]. Despite the outstanding precision and reliability of conventional analytical methods such as chromatography and mass spectrometry for the detection of small molecules, their applications remain limited by several inherent drawbacks. These techniques typically rely on costly and bulky instrumentation, require labor-intensive and time-consuming sample preparation, and demand highly trained personnel for operation. Such constraints hinder their implementation in point-of-care testing (POCT) and restrict their capacity to deliver real-time monitoring or support rapid clinical decision-making. Furthermore, the lack of portability makes these methods unsuitable for resource-limited settings or on-site detection scenarios. In contrast, electrochemical sensors, with their inherent advantages of rapid response, facile miniaturization, and integration potential, are uniquely positioned to bridge this gap. They not only overcome the limitations of conventional techniques but also serve as an indispensable platform for the development of next-generation detection systems, particularly in POCT, environmental monitoring, and personalized medicine. As an important analytical technique, electrochemical strategies offer a convenient, rapid, cost-effective, and efficient solution for the detection of biological small molecules, demonstrating significant potential in the analysis of small molecular biological samples in basic biomedical research, clinical diagnosis and pharmaceutics, including drug screening, pharmaceutical quality control and the evaluation of nanoformulations.

This review systematically summarizes recent research progress in the electrochemical detection of biological small molecules such as dopamine, glucose, uric acid, hydrogen peroxide, glutathione, lactic acid and cholesterol (Table 1). This study goes beyond the perspective of previous reviews, which are often limited to a single biomarker, a specific sensing strategy, or a single class of nanomaterials, and establishes a systematic review approach oriented toward practical applications in biomedical and pharmaceutical analysis. This approach enables us to span different biomarkers and perform horizontal comparisons and integrated evaluations in terms of sensing principles, nanomaterial design strategies, and practical considerations for reliable detection in complex biological matrices. By providing an in-depth analysis of existing technologies, it highlights current challenges and outlines future development directions. In doing so, this review aims to offer systematic technical insights and innovative ideas for researchers working at the interface of biomedical science, biotechnology and nanotechnology, thereby promoting the iterative upgrading of interdisciplinary detection technologies and accelerating their large-scale application in clinical diagnosis, basic biomedical research and nanomedicine translation. Therefore, such sensing platforms hold promise not only for diagnostic purposes but also for pharmacokinetic studies, drug safety evaluation, and the development of targeted nanomedicines. Such sensing platforms are also promising for pharmacokinetic investigations, drug safety assessment, and the development of targeted nanomedicines.

**Table 1:** Performance comparison of representative electrochemical sensors for small-molecule analytes.

Analytes	Sensing Materials	Methods	Liner Range (µM)	LOD (µM)	Ref.
Dopamine	Cu-TCPP/graphene/GCE	DPV	0.02–100 100–1000	0.0036	[8]
	p-L-Trp/GN/GCE	DPV	0.2–100	0.06	[9]
	Nafion/rGO/CSF	DPV	0.001–30	0.001	[10]
	N-GQDs/GCE	CV, LSV	0.001–1000	0.00015	[11]
	CuO-MgO NC	CV, I-t	10–100	6.4	[12]
Glucose	SNF/RGO/GOx	I-t	0.3–100	0.3	[13]
	Ru(dmo–bpy)_2_Cl_2_/GDH/PDAMWCNT/SPCEs	CV	100–30,000	94	[14]
	GRE/PtCo/GOx/Nafion	I-t	40–218	21	[15]
	Ni/Ni O/NC/GCE	I-t	0.6–860	0.2	[16]
	Ni-BDC-NH2	I-t	10–1400	3.82	[17]
	(XSBR-PEDOT:PSS-AMWCNTs/AuNPs/SPE)	CV	50–600	3.2	[18]
Uric Acid	CuO/GCE	CV	1–35,100	0.6	[19]
	UOx/Fc/Cu_2_O/GCE	DPV	10–1000	0.0596	[20]
	GNSs/CC	DPV	20–1000 0.5–20; 0.5–20	0.31 (AA) 0.01 (DA) 0.03 (UA)	[21]
	PVP-GR/GCE	LSV	4.0–1000 0.02–0.2; 0.2–100 0.04–1.0; 1.0–100	0.8 (AA) 0.002 (DA) 0.02 (UA)	[22]
	GR-MWCNT/GCE	DPV	100–1000 5–50 50–500	6.71(AA) 0.58(DA) 7.30(UA)	[23]
	HNGA/GCE	DPV	50–1500 5–50 5–50	16.7 (AA) 0.22 (DA) 0.12 (UA)	[24]
H_2_O_2_	Cu_2_O/AuCu/Cu	I-t	0.3-10	0.14	[25]
	Pt/MoSe_2_	I-t	8–6818	2.56	[26]
	Au_3_Pt_7_/Co-MOFs/GCE	I-t	0.1–5000 5000–60,000	0.02	[27]
	AgNPs/rGO/GCE	CV	1–276	0.18	[28]
	Ag-CeO_2_/Ag_2_O/GCE	CV, I-t	0.01–500	6.34	[29]
	COFTZT-DVA/CNT@PB/GCE	I-t	2.38–1050	0.79	[30]
Lactic acid	MoS_2_-AuPt	SWV	5–3000	0.33	[31]
	Co_3_O_4_/CuO@MWCNTs NCs	CV	0.001–100,000	0.000055	[32]
	Pt@ Chitosan/ZnTiO_3_NCs/GCE	DPV	300–12,000	22.36	[33]
	Cu-TCPP(Fe)/Au/LOx	CV	0.000013–100,000	0.00000091	[34]
	Lox @ CS PC	CV	10–35,000	0.144	[35]
Cholesterol	ChOx-Chit/PB-PEDOT/Au-Ag@Au NPs/SPCE	I-t	10–1000	3.3	[36]
	CoFe_2_O_4_@MoS_2_/Au-ChOx	DPV	5–100	0.09	[37]
	ChOx/MoSe_2_/CNTs	CV	0–100	0.082	[38]
	PIND/CuNPs/PGE	CV, SWV	0.015–0.195	0.00498	[39]
	NiO/CuO/GCE	CV	800–65,000	5.9	[40]
	C-pept/PLA NM/SPE	EIS	2–6	6.31	[41]
Glutathione	AuNP-PEDOT/GCE	I-t	0.5–10 3000–15,000	0.173	[42]
	Ag-MOF	DPV	0.0001–1	0.000018	[43]
	CuNPs@ NPC/GCE	DPV	0.0001–10	0.000067	[44]
	Cu(II)-PMMS/AgNPs/PEG-OH/Chit	SWV	0.1–125	0.03	[45]
	mTiO_2_/Ag_2_S	PEC	10–10,000	6.39	[46]
	GO-SiO_2_@AgNPs	I-t	0.25–3	0.17	[47]

## 2. Electrochemical Sensor

**Figure 1:**
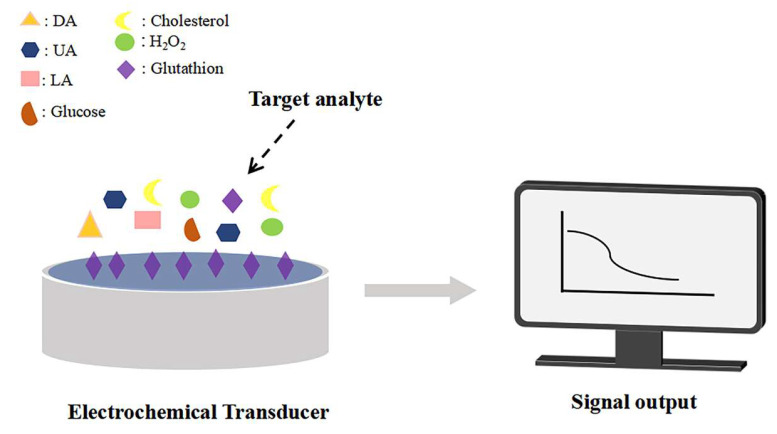
Schematic illustration of the working principle of electrochemical sensors.

Electrochemical sensors are highly integrated standalone devices that can transduce physicochemical cues into quantifiable electrical signals that can be detected and analyzed. Their structure mainly consists of three major parts: the biorecognition element, the signal transducer, and the signal processor [48]. The working principle of electrochemical sensors is based on the specific recognition interactions between the target analyte and the recognition element, as well as the physical or chemical changes triggered by this binding. When the target analyte binds to the recognition element, the signal transducer converts this interaction into amperometric response correlating with analyte concentration that is related to the concentration of the target analyte. Subsequently, the signal processor collects and amplifies this current signal, ultimately outputting an electrical signal that can be used for quantitative analysis, thereby enabling the detection and analysis of the target substance [49] (Figure 1). In electrochemical sensing applications, the most widely adopted electrode configuration is the three-electrode (3E) platform, comprising the working electrode (WE), counter electrode (CE), and reference electrode (RE). The three-electrode configuration constitutes the cornerstone for a spectrum of electrochemical characterization techniques, with cyclic voltammetry (CV), electrochemical impedance spectroscopy (EIS), and differential pulse voltammetry (DPV) being the most widely employed (Table 2). These techniques enable the quantification of electrode reactions, interfacial properties, and target analyte concentrations through the analysis of electrochemical signals, including current, potential, and impedance.

### 2.1. Cyclic Voltammetry (CV)

Cyclic voltammetry is one of the most widely used electrochemical analysis techniques. Its working principle is based on imposing a linearly sweeping potential to the working electrode and registering the concomitant faradaic current. This strategy involves applying an isosceles triangular pulse voltage to the working electrode (the potential is linearly scanned from the initial value to the final value and then reversed back to the initial value), while recording the current–potential curve [50]. When the potential is scanned from the initial value in one direction, electroactive species on the electrode surface undergo oxidation or reduction reactions, generating corresponding oxidation or reduction currents. For example, when the potential reaches the oxidation potential of a species, it is oxidized, producing an oxidation current; conversely, when the potential is scanned back to the reduction potential, the previously oxidized species is reduced, generating a reduction current. By analyzing the characteristics of the current–potential curve (e.g., peak current, peak potential, etc.), thermodynamic and kinetic information about the electrode reaction can be obtained, including reaction reversibility, electron transfer number, and reaction rate.

Cyclic voltammetry is widely used in studies of electrochemical reaction mechanisms, electrode material characterization, and biosensor development due to its simplicity and ability to provide rich electrochemical information. However, CV also has certain limitations, particularly in complex platforms where multiple redox reactions may overlap, making data interpretation challenging. Therefore, in practical applications, CV is often combined with other electrochemical techniques (e.g., electrochemical impedance spectroscopy, differential pulse voltammetry, etc.) to comprehensively elucidate reaction mechanisms and improve the accuracy and reliability of the analysis.

### 2.2. Electrochemical Impedance Spectroscopy (EIS)

Electrochemical Impedance Spectroscopy (EIS) is a highly efficient and sensitive electrochemical characterization technique capable of rapidly evaluating the interfacial properties and reaction kinetics of electrochemical platforms. This strategy applies an alternating current (AC) signal with varying frequencies (typically a sinusoidal waveform) to the electrochemical platform and measures the ratio of the platform’s voltage response to the applied current, thereby obtaining the electrochemical impedance [51]. Electrochemical impedance is typically represented in complex form. In EIS experiments, the frequency range of the AC signal usually spans from high frequencies to low frequencies. A complex impedance spectrum, usually in the form of a Nyquist plot or Bode plot, can be constructed by systematically varying the frequency and measuring the corresponding impedance values. As highlighted in Electrochemical Impedance Spectroscopy by Orazem and Tribollet, the impedance spectrum exhibits distinct features across different frequency domains. The high-frequency region predominantly reflects the ionic transport properties of the electrolyte, while the low-frequency region is governed by charge transfer processes at the electrode interface. Such frequency-dependent impedance behavior provides critical insights into reaction kinetics and interfacial mechanisms [52]. Through in-depth analysis of the electrochemical impedance spectrum, a variety of key information about the electrochemical platform can be obtained. For example, by fitting the impedance spectrum data with an equivalent circuit model, parameters such as charge transfer resistance, double-layer capacitance, and diffusion impedance can be quantified, enabling the evaluation of the electrochemical performance metrics, reaction kinetics, and interfacial stability of materials. Additionally, EIS technology offers the advantage of real-time monitoring, allowing for the dynamic tracking of changes in the electrochemical platform, which provides important dynamic information for the study of electrode reaction processes.

In summary, electrochemical impedance spectroscopy as an important electrochemical characterization technique, not only provides profound insights into interfacial properties, reaction mechanisms, and performance optimization pathways of electrochemical platforms, but also offers robust theoretical support and experimental evidence for electrochemical research and applications. Notably, EIS is particularly advantageous for probing charge transfer resistance at sensor interfaces, and it is frequently employed to evaluate the immobilization efficiency of enzyme-based sensors. These capabilities have promoted its widespread application in diverse fields, including materials science and sensor development.

### 2.3. Differential Pulse Voltammetry (DPV)

DPV is a highly sensitive electrochemical analysis technique based on superimposing a series of small rectangular pulses on a slowly linearly varying substrate potential, the voltammetric response is obtained by measuring the difference between the current before and after the pulse is applied. Specifically, DPV applies a constant-amplitude potential pulse to the baseline potential and records the current response just prior to the conclusion of each pulse. The short duration of these potential pulses significantly diminishes the impact of background current, while effectively amplifying the faradaic current associated with the redox reactions of electroactive species. By calculating the current difference before and after the pulse, DPV enables effective suppression of capacitive background currents, thereby yielding an enhanced signal-to-noise ratio (SNR) volt–ampere curve. Owing to this advantage, DPV achieves excellent detection sensitivity and low detection limits, making it particularly suitable for the quantitative analysis of low-concentration samples.

Furthermore, the superior SNR and differential current measurement mechanism of DPV make it especially advantageous for detecting trace small molecules in complex sample matrices. For example, Zhang et al. applied a DPV-based method using modified electrodes to simultaneously detect ascorbic acid, dopamine, and uric acid in mixed systems, achieving well-resolved and distinguishable oxidation peaks despite their closely overlapping redox potentials [53]. In a similar study, Wei et al. employed Pd nanoparticle/reduced graphene oxide-modified electrodes to detect the same analytes in real biological samples, demonstrating excellent anti-interference performance [54]. These studies demonstrate that DPV maintains high selectivity in complex sample environments, establishing it as a preferred strategy for trace-level small-molecule analysis in such platforms. Its robust performance and adaptability highlight its significant potential for broad applications in biosensing, clinical diagnostics, and environmental monitoring.

### 2.4. Commonly Used Electrode Materials

The selection of electrode materials plays a pivotal role in determining the sensitivity, selectivity, and stability of electrochemical sensors. A systematic understanding of the intrinsic electrochemical properties of commonly employed materials provides the theoretical foundation for the rational design of sensing platforms for biological small molecules. Nanomaterials, owing to their unique physical and chemical properties, exhibit significant advantages in the construction of electrochemical sensing interfaces. They play an irreplaceable role in enhancing electrode conductivity, electrocatalytic activity, and surface reaction kinetics. Therefore, in the development of novel electrochemical sensors, nanomaterials are widely used in electrode modification and functional interface engineering, mainly including the carbon-based materials, metals and metal oxides, metal–organic frameworks (MOFs), and conductive polymers.

#### 2.4.1. Carbon-Based Materials

Carbon-based materials are among the most widely employed electrode materials in electrochemical sensors owing to their outstanding electrical conductivity, large specific surface area, chemical stability, and facile surface functionalization. Their unique physicochemical properties enable efficient electron transfer, effective adsorption, and immobilization of biomolecules, thereby significantly enhancing sensor performance.

Graphene, as a two-dimensional, atomically thin carbon sheet, provides an ultrahigh carrier mobility and an extensive π-conjugated network that facilitates rapid charge transfer and strong interactions with target analytes [55]. Carbon nanotubes (CNTs), on the other hand, offer one-dimensional conductive pathways, high aspect ratios, and remarkable mechanical robustness, which contribute to excellent structural integrity and electrochemical durability. Furthermore, the inherent chemical inertness of carbon-based nanomaterials imparts high stability under repeated redox cycling, making them suitable for long-term operation [56].

Moreover, the versatility of carbon-based materials enables their hybridization with metals, metal oxides, and polymers to form multifunctional composites that integrate conductivity, catalytic activity, and biocompatibility. Such hybrid systems have shown remarkable improvements in lowering detection limits and enhancing selectivity. Looking ahead, the rational design of carbon-based nanostructures with controlled morphology, tailored surface chemistry, and defect engineering is expected to further enhance the performance of electrochemical sensors and lay a solid foundation for their practical applications in point-of-care diagnostics, environmental monitoring, and food safety testing.

#### 2.4.2. Metals and Metal Oxides

Metals and metal oxides are widely employed as electrode materials in electrochemical sensors due to their excellent electrical conductivity, intrinsic catalytic activity, and structural diversity. Noble metals such as gold, platinum, and silver exhibit outstanding electrocatalytic performance and biocompatibility, making them highly effective for the sensitive detection of biomolecules and facilitating electron transfer processes at the electrode–electrolyte interface. However, the high cost and scarcity of noble metals restrict their large-scale applications.

To address these limitations, transition metal oxides (TMOs) including MnO_2_, Fe_3_O_4_, Co_3_O_4_, and NiO have been extensively investigated as cost-effective alternatives. Their abundant redox-active sites, multiple oxidation states, and tunable nanostructures collectively enhance electrocatalytic performance and promote effective interaction with target molecules.

For instance, a recent study demonstrated that acetate-assisted in situ electrodeposited β-MnO_2_ nanosheets exhibit high sensitivity (461.87 µAM^−1^ cm^−2^) for non-enzymatic glucose detection over a wide concentration range (1 µM to 1 mM), with strong selectivity and good stability in complex electrolyte environments [57]. In addition to MnO_2_, Fe_3_O_4_-based materials have shown excellent performance in neurotransmitter and metabolite sensing. Gaya et al. [58] demonstrated that Fe_3_O_4_ nanoparticles combined with Vulcan carbon and Nafion enabled simultaneous determination of dopamine, uric acid, and ascorbic acid with clear peak separation and low detection limits. Collectively, these findings underscore the broad application potential of transition metal oxides in diverse detection scenarios. As multifunctional and cost-effective materials, they offer a robust platform for the development of high-performance electrochemical sensors. Furthermore, they provide essential theoretical foundations and practical guidance for the rational selection of electrode materials in subsequent small-molecule detection.

#### 2.4.3. Metal–Organic Frameworks (MOFs)

Metal–organic frameworks (MOFs) are crystalline porous materials constructed from metal nodes and organic linkers, exhibiting large surface areas, tunable pore sizes, and high chemical versatility. These structural advantages endow MOFs with great potential in electrochemical sensing, where porous frameworks facilitate analyte diffusion and abundant active sites enable strong host–guest interactions [59]. Nevertheless, the intrinsically low electrical conductivity of pristine MOFs has limited their standalone applications in electrochemical devices.

To overcome this issue, researchers have developed MOF-based composites by integrating conductive materials such as graphene, carbon nanotubes, conducting polymers, or metal nanoparticles, which significantly improve electron transport and catalytic activity [60]. Another effective strategy is the preparation of MOF-derived materials (e.g., porous carbons, metal oxides, or hybrid nanostructures), which retain the high surface area of MOFs while providing enhanced conductivity and stability [61]. For instance, Kumar et al. [62] comprehensively reviewed recent progress in MOF-based biosensors and reported their successful applications in detecting glucose, dopamine, hydrogen peroxide, ascorbic acid, and uric acid. They emphasized that MOF-derived composites and functionalized MOFs markedly improved sensor stability and reduced background current in complex biological matrices. In another work, Gu et al. [63] developed a MOF-5@MWCNT composite electrode that combined the large surface area of MOF-5 with the excellent conductivity of MWCNTs to synergistically enhance sensing performance. The sensor enabled the simultaneous determination of acetaminophen and dopamine, exhibited outstanding analytical performance in real sample analysis, and maintained high selectivity even in the presence of common interfering species. Collectively, these findings underscore the versatility of MOFs and their derivatives, confirming their potential as multifunctional and cost-effective electrode materials for high-performance electrochemical sensors. Their structural tunability, combined with rational composite or derivative design, provides a robust foundation for small-molecule detection in complex biological and environmental systems.

However, despite the significant performance advantages of these nanomaterials, their potential toxicity and the environmental footprint of their synthesis have not yet been fully clarified and remain key constraints on large-scale and practical deployment. On the one hand, material composition, particle size and morphology, surface chemistry, and metal-ion leaching behaviour can critically modulate cytotoxicity, oxidative-stress-related biological responses, and long-term bioaccumulation risks in organisms and environmental compartments. On the other hand, conventional preparation of nanomaterials often relies on organic solvents, strong reducing agents, and high-temperature/high-pressure, energy-intensive conditions, which can generate hazardous waste and thereby adversely affect environmental safety. In response to these issues, an increasing number of studies have proposed green strategies, such as using water or other low-toxicity solvents, plant-extract- or microbial/biomass-derived reducing agents and stabilizers, and bio-based or biodegradable substrates to reduce the use of toxic reagents and process-related emissions [64]. At the device level, constructing composite architectures in which active nanomaterials are tightly immobilized or encapsulated within carbon materials, polymers, MOFs, or hydrogel matrices can further suppress metal leaching and reduce direct contact with biological systems while maintaining electrocatalytic performance [65]. Therefore, the design of nanomaterial-modified electrodes in the future should pursue high sensitivity and selectivity while simultaneously incorporating safe- and green-by-design principles to move these sensing platforms closer to real-world application.

## 3. Electrochemical Analysis of Small Molecules

### 3.1. Dopamine (DA)

Dopamine is an important organic compound belonging to the catecholamine and phenethylamine families, as well as a key neurotransmitter in the central nervous platform. It plays a crucial role in regulating various physiological processes in the human body, including metabolism, immune responses, memory formation, sleep regulation, and cognitive functions [66]. The monitoring of dopamine levels in the human body is crucial, and its deficiency or dysregulation can lead to the symptoms of many diseases, such as Parkinson’s disease and cardiovascular disease [67]. Consequently, real-time monitoring of dopamine levels holds significant implications for early disease diagnosis and therapeutic interventions. The development of a simple, efficient, highly sensitive and highly selective strategy for the detection of dopamine has become an important research direction in the biomedical field. Currently, the detection of dopamine mainly relies on traditional strategies such as high-performance liquid chromatography (HPLC) [68] and spectrophotometry [69]. However, these strategies often face the limitations of insufficient sensitivity, poor selectivity and complex operation in the detection of very low concentrations of dopamine [70]. To overcome these limitations, researchers have shifted their focus to electrochemical sensing technologies. By developing specific electrochemical sensors based on biomolecular recognition, they have achieved rapid, real-time, and highly selective detection of dopamine [71].

**Figure 2:**
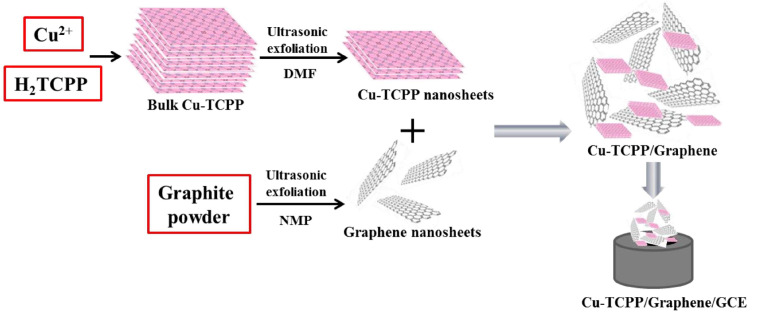
Schematic plot of the construction of Cu-TCPP/graphene/GCE. Reprinted with permission from Ref. [8]. Copyright © 2023 by the authors.

In recent years, significant progress has been made in the development of electrochemical dopamine sensors. Researchers have significantly improved the sensitivity, selectivity and stability of the sensor by optimizing the electrode materials, refining surface functionalization strategies and optimizing the detection conditions. In this part, the research progress and application prospect of dopamine electrochemical sensor in recent years will be introduced (Table 3). Ji et al. [8] developed an ultrasensitive dopamine sensor by modifying a glassy carbon electrode with a layered Cu-TCPP (2D MOF)/graphene composite. The synergistic interface increased electroactive area, accelerated charge transfer, and enhanced DA accumulation/catalytic kinetics, enabling DPV detection with a wide dynamic range and nanomolar LOD, and the method was validated in serum via standard addition (Figure 2). However, the long-term anti-pollution stability still lacks systematic evaluation. If a broader range of real clinical samples could be included for validation, it would be more beneficial in supporting its translational application. Gong et al. [9] constructed a highly sensitive electrochemical sensor for dopamine detection via electropolymerization of L-tryptophan onto a graphene-modified glassy carbon electrode by electrochemical polymerized L-tryptophan (p-L-Trp) on a graphene-modified glassy carbon electrode, which has been successfully applied to the detection of dopamine, the sensor exhibited excellent electrocatalytic activity for the oxidation of DA with a detection limit as low as 0.06 µM. However, graphene-based electrodes often suffer from poor dispersion and aggregation issues, which may compromise the reproducibility of the sensor performance. Additionally, the electropolymerization process requires precise control of potential and time, limiting its scalability for large-scale fabrication. And Ji et al. [10] investigated a flexible organic electrochemical transistor (OECT) sensor based on carbonized silk fabric (CSF) wrapped with Nafion and reduced graphene oxide (rGO). The hierarchical structure of this electrode effectively improves the conductivity of the electrode and prevents the aggregation of RGO and Nafion, thus achieving an extremely low limit of detection (LOD) (1 nM) and a wide detection range (1–30 nM) for DA, which can be used to improve the sensitivity of the electrode, it exhibits great potential in the field of flexible electronics. While the flexibility and low LOD are promising for wearable applications, the stability and consistent performance of such organic transistors under long-term physiological conditions (e.g., variable pH, mechanical stress) require further investigation for practical deployment.

Thadathil’s research team synthesized N-GQDs by a simple one-step hydrothermal strategy and utilized them as electrochemical catalysts. The nitrogen-doped graphene lattice in the N-GQDs significantly enhanced their electrocatalytic activity for DA, and they can detect DA as low as 0.15 nM, which was successfully and efficiently achieved for DA detection [11]. Moreover, Paramparambath et al. [12] successfully developed CuO-MgO nanocomposites and systematically evaluated their dopamine-sensing performance by cyclic voltammetry and chronoamperometry. The porous CuO-MgO architecture affords a high specific surface area, abundant adsorption sites and efficient charge–transport pathways, thereby enhancing the oxidation current and improving sensitivity toward dopamine in complex matrices. In artificial sweat, the CuO-MgO-based sensor exhibits excellent sensitivity, selectivity, rapid response and good stability, suggesting that this nanocomposite is a promising sensing material for integration into wearable devices for the early diagnosis and real-time monitoring of neurological disorders such as Parkinson’s disease and depression. Furthermore it is recommended that the performance in real human sweat (with varying pH values, electrolyte compositions, and interfering substances) be further validated.

These studies not only propel the advancement of dopamine sensing technologies, but also provide new possibilities for the application of flexible electronics and wearable devices. In the future, with continued advances in materials science and sensing technologies, efforts should be directed toward optimizing and standardizing fabrication protocols to improve scalability and batch-to-batch reproducibility, as well as conducting rigorous validation in clinical samples to comprehensively assess real-world analytical performance in complex matrices.

**Table 3:** Performance comparison for DA detection with different sensors.

Sensing Materials	Methods	Linear Range (µM)	LOD (µM)	Ref.
Cu-TCPP/graphene/GCE	DPV	0.02–100, 100–1000	0.0036	[8]
p-L-Trp/GN/GCE	DPV	0.2–100	0.06	[9]
Nafion/rGO/CSF	DPV	0.001–30	0.001	[10]
N-GQDs/GCE	CV, LSV	0.001–1000	0.00015	[11]
CuO-MgO NC	CV, I-t	10–100	6.4	[12]

### 3.2. Glucose

Glucose is a crucial biomolecule that sustains the normal life processes of humans. However, its excessive accumulation in the bloodstream can result in metabolic disturbances, ultimately leading to the onset of diabetes [72]. Currently, diabetes has become one of the largest and most important diseases affecting human health worldwide. Therefore, accurate, rapid and efficient detection of glucose in blood is very important for the diagnosis and treatment of diabetes [73]. Compared with the traditional detection strategies, the electrochemical strategyis still considered to be the most convenient, effective and promising strategydue to its high sensitivity, convenient operation and simple instrument.

**Figure 3:**
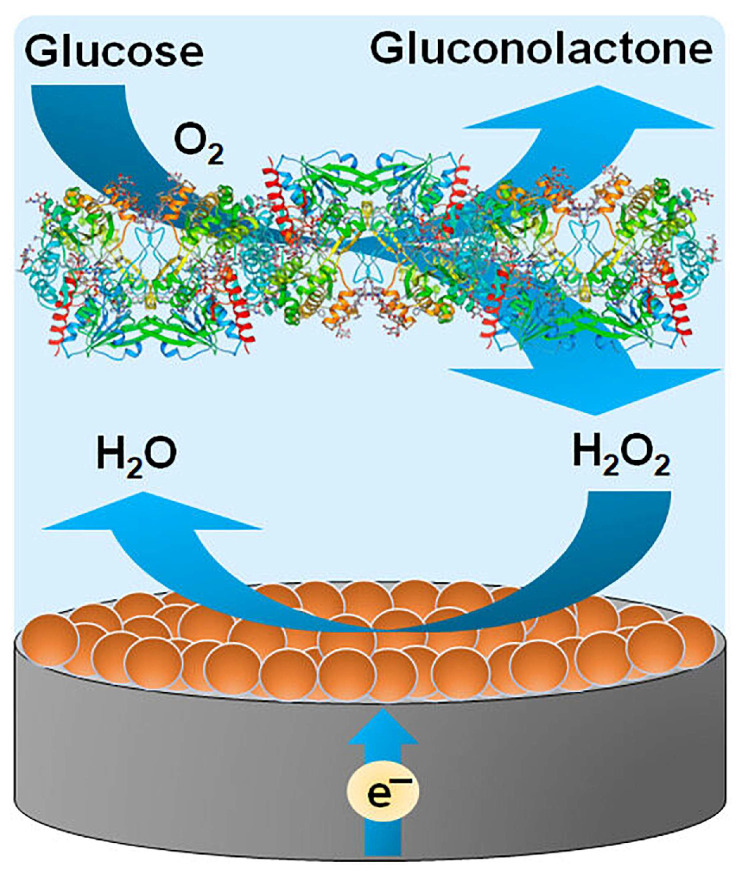
Schematic illustration of the operating principle of the amperometric enzyme–nanozyme glucose biosensor. Reprinted with permission from Ref. [15]. Copyright © 2025 by the authors.

Glucose electrochemical sensors are mainly divided into two types: enzymatic and non-enzymatic. Enzymatic sensors that employ specific biocatalysts, such as glucose oxidase or glucose dehydrogenase, typically provide high intrinsic selectivity and have been widely adopted in commercial test strips and point-of-care devices. For instance, Chen et al. [13] developed a silk-based enzymatic electrochemical sensor for sweat glucose by integrating silk nanofibrils (SNF) with reduced graphene oxide (RGO) and glucose oxidase (GOx) on a screen-printed three-electrode platform. In this design, the silk matrix facilitated efficient enzyme immobilization and enhanced interfacial electron transfer, enabling stable glucose detection with improved tolerance to common interferents. The feasibility of the sensor was further validated using human sweat samples collected under controlled conditions. Jeon et al. [14] reported an electrochemical glucose biosensor using flavin adenine dinucleotide (FAD)-dependent glucose dehydrogenase (FAD-GDH) to reduce the oxygen dependence typical of GOx-based systems. By introducing polydopamine-functionalized MWCNTs, they achieved crosslinker-free co-immobilization of the Ru(dmo-bpy)_2_Cl_2_ mediator and the enzyme on screen-printed electrodes, improving interfacial stability. The sensor showed selective glucose detection against common electroactive interferents and demonstrated feasibility in serum spiking tests, supporting the practical value of this interface-engineering strategy. Kausaite-Minkstimiene et al. [15] developed an amperometric enzyme–nanozyme glucose biosensor by layer-by-layer assembling PtCo bimetallic nanoparticles, glucose oxidase (GOx), and a Nafion film on a graphite rod electrode. The PtCo nanozyme promotes the electrochemical reduction of Gox-generated H_2_O_2_, thereby converting the enzymatic reaction into a glucose-dependent current signal. The sensor showed good repeatability, tolerance toward common interferents, and short-term operational/storage stability, and its feasibility was demonstrated in serum samples after dilution into the working range (Figure 3).

Despite these advantages, enzymatic sensors may still suffer from enzyme-related limitations, including sensitivity to fluctuations in temperature, pH, and chemical environments, as well as added complexity and cost associated with enzyme immobilization and storage. In contrast, non-enzymatic sensors rely on direct electrocatalysis at the electrode surface and are attractive for simplified fabrication, improved operational robustness, and scalable manufacturing, making them promising for miniaturized and wearable monitoring. Therefore, non-enzyme glucose sensors, which are low-cost, fast-responding, stable, and have high sensitivity, have gradually become a research focus [74,75]. Nevertheless, their performance in complex biological matrices can be compromised by coexisting electroactive interferents and biofouling. Achieving reliable practical deployment thus often requires selective and antifouling interface engineering, together with rigorous validation in real samples. So far, researchers have made significant efforts in developing accurate, fast, and low-cost glucose sensors, and have fabricated a range of materials that promote electron transfer and catalytic performance metrics, such as carbon materials0, metals [76] and metal oxides [77,78,79] among others. Currently, a variety of electrochemical sensors based on different materials and principles have been successfully applied to glucose detection (Table 4).

In the research of non-enzyme glucose sensors, the selection and design of materials play a crucial role in the performance metrics of the sensor. Liang et al. [16] successfully synthesized nitrogen-doped porous carbon foams enriched with Ni and NiO nanoparticles through a sacrificial template strategy and used them as electrode materials to construct a non-enzyme electrochemical glucose sensor. The sensor leverages the synergistic catalytic effect of Ni and NiO, significantly enhancing detection sensitivity, with a low detection limit of 0.2000 µM, a wide detection range from 0.6000 µM to 8.600 mM This metal and metal oxide composite electrocatalyst, through the mutual cooperation and synergistic catalytic effect between the metal and metal oxide, can significantly improve its electrochemical performance metrics, demonstrating superior catalytic activity and stability.

Metal–organic frameworks are porous crystalline materials self-assembled from metal clusters and organic linkers. Due to their unique structural characteristics, MOFs have demonstrated significant advantages in the field of glucose detection. MOF materials possess precisely tunable pore structures, ultra-high specific surface areas, and a rich distribution of active sites. These structural features not only facilitate electron transfer but also significantly augmented electrical conductivity and electrochemical adsorption capacity, making them an ideal catalytic platform for glucose sensing [80,81]. Daud et al. [17] developed a non-enzyme glucose sensor based on metal–organic frameworks, providing an efficient alternative for long-term blood glucose monitoring. They developed a Ni-based MOF sensor using a solvothermal strategy, reacting Ni^2+^ with BDC or its derivatives (BDC-NH_2_ and H_2_BDC-OH). The study revealed that the multidimensional porous structure and hierarchical arrangement of Ni-BDC-NH_2_ significantly accelerated glucose diffusion, delivering superior electrochemical performance metrics. Despite these merits, MOF-based sensors may face practical challenges related to framework stability, structural evolution during long-term electrochemical polarization, and potential metal-ion leaching, all of which can affect signal drift and biocompatibility. Therefore, beyond short-term performance metrics, long-duration operation tests and stability verification in realistic matrices are needed to better support practical use.

In recent years, with the rapid development of material science and microelectronics technology, wearable sensors have made significant progress in glucose detection. These sensors primarily monitor glucose concentrations in real time from sweat, saliva, or interstitial fluid using non-invasive or minimally invasive strategies. Chen et al. [18] introduced functionalized gold nanoparticles (AuNPs) into amine-functionalized multi-walled carbon nanotubes (AMWCNTs) as efficient catalysts, and crosslinked them with carboxylated butadiene styrene rubber (XSBR) and PEDOT: PSS. Subsequently, the composite material was integrated into screen-printed electrodes (SPE) to develop a novel wearable, non-invasive electrochemical sensor (XSBR-PEDOT: PSS-AMWCNTs/AuNPs/SPE). This sensor, especially in low glucose concentration environments, exhibited a wide linear detection range and was able to accurately detect subtle changes in glucose concentration. In summary, significant progress has been made in electrochemical glucose detection research. With the deep integration of nanotechnology and material science, electrochemical glucose sensors are poised to provide strong support for the early diagnosis and precise treatment of diseases such as diabetes.

In conclusion, electrochemical glucose sensors have made significant strides through the development of advanced functional materials, including metal oxides, metal–organic frameworks, and hybrid nanocomposites, leading to significant improvements in sensitivity, stability, and suitability for continuous monitoring. Nonetheless, future research should go beyond material-level performance optimization and place greater emphasis on system-level integration, including antifouling interface engineering, flexible and biocompatible device architectures, and robust calibration strategies. Such interdisciplinary efforts are critical to translating lab-scale technology into reliable, smart, and affordable wearable glucose monitoring systems for patients with large diabetes.

**Table 4:** Performance comparison for Glucose detection with different sensors.

Sensing Materials	Methods	Linear Range (µM)	LOD (µM)	Ref.
SNF/RGO/GOx	I-t	0.3–100	0.3	[13]
Ru(dmo–bpy)_2_Cl_2_/GDH/PDAMWCNT/SPCEs	CV	100–30,000	94	[14]
GRE/PtCo/GOx/Nafion	I-t	40–218	21	[15]
Ni/Ni O/NC/GCE	I-t	0.6–860	0.2	[16]
Ni-BDC-NH_2_	I-t	10–1400	3.82	[17]
(XSBR-PEDOT:PSS-AMWCNTs/AuNPs/SPE)	CV	50–600	3.2	[18]

### 3.3. Uric Acid (UA)

Uric acid (UA), a key product of purine metabolism in the human body, is widely present in biological fluids such as serum and urine [82]. Abnormal UA levels are important indicators for various diseases, including gout, pneumonia, hyperuricemia, and leukemia [83]. Therefore, rapid and accurate detection of UA concentration is crucial for early disease diagnosis and physiological monitoring. In recent years, various UA detection strategies have been developed and applied in clinical and research fields, including spectroscopic techniques [84], electrochemical strategies [85], capillary electrophoresis [86], chemiluminescence [87] and chromatograph [88]. However, these traditional strategies still have some limitations, such as time consumption, complex sample preparation, and high costs. In contrast, electroanalytical strategies, with their reliability, cost-effectiveness, simplicity, and high sensitivity, exhibit immense promise for detecting UA at low concentrations (Table 5).

Various electrochemical sensors based on different materials and principles have been successfully applied to uric acid detection. Among them, nanomaterials, such as metal and metal oxide nanoparticles, have been effectively used to construct electrochemical and biosensors due to their unique chemical and physical properties. Nanostructures provide critical functionalities for sensing devices, including enhanced catalytic properties in electrochemical sensing, promotion of electron transfer between analytes and electrode surfaces, immobilization and labeling of biomolecules, and their role as reactants [89,90,91]. Among these metal oxide nanoparticles, CuO nanostructures have garnered significant attention due to their distinctive physicochemical properties, offering broad application prospects in fields such as chemical sensors, magnetic storage media, catalysts, sensors, and semiconductors. Recently, Buledi et al. [19] synthesized CuO nanostructures using sodium hydroxide as a reducing agent through an aqueous chemical growth strategy. These CuO nanostructures demonstrated remarkable electrocatalytic behavior in UA detection. The sensor achieves a robust and linear electrochemical response over a wide range of urine concentrations and improves sensitivity. Beyond CuO, copper-oxide-derived architectures have also been explored to further enhance UA sensing. For example, Yan et al. [20] developed a highly sensitive enzymatic uric acid electrochemical biosensor by constructing a multilayer UOx/Fc/Cu_2_O interface on a glassy carbon electrode, where Cu_2_O NPs provides an electroactive, chemical activator (Fc) serves as an electron mediator to accelerate interfacial charge transfer, and uricase (UOx) affords molecular specificity toward UA. Meanwhile, the sensor demonstrated good stability and high selectivity for UA in the presence of potential interferents, highlighting its strong potential for reliable UA detection and its promise for future clinical settings.

Generally, ascorbic acid, dopamine, and uric acid are three biomolecules that commonly coexist in biological fluids and play crucial roles in human physiological metabolism. Therefore, developing a strategy capable of simultaneously and sensitively detecting these three biomolecules holds significant practical importance. Graphene (GN) electrodes have been recognized as a potential new nanomaterial for sensor design due to their excellent electrical conductivity, high ductility, high thermal conductivity and large specific surface area [92]. Although graphene electrodes possess many outstanding properties, their nanoscale thickness limits their standalone application. To address this issue, researchers have developed various graphene-based composite platforms. Among them, carbon cloth (CC), as an excellent electronic substrate, can be effectively integrated with flexible electronic devices to enable biochemical signal sensing and detection [93,94]. Meng et al. [21] successfully fabricated a GNSs/CC flexible sensor by directly growing three-dimensional (3D) graphene nanosheets (GNSs) on CC substrates using thermal chemical vapor deposition (CVD) technology. This structure ensures strong binding between GN and CC fibers, avoiding the detachment issues of physical adsorption strategies while leveraging the flexibility of CC and the high chemical activity and large specific surface area of 3D GN. The sensor exhibited excellent catalytic activity for the simultaneous detection of AA, DA, and UA. From a practical application point of view, however, CVD growth can be equipment- and energy-intensive, and performance should be further corroborated under repeated bending/stretching cycles and biofouling-prone environments to confirm durability for wearable applications.

**Figure 4:**
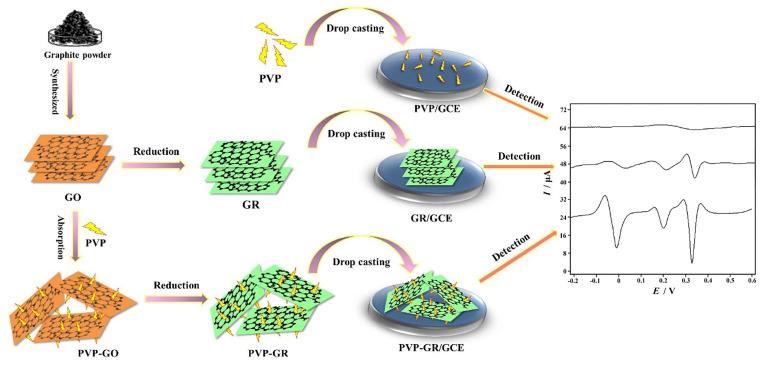
Diagram for the preparation of PVP/GCE, GR/GCE and PVP–GR/GCE. Reprinted with permission from Ref. [22]. Copyright © 2020 by the authors.

To further improve the performance metrics of graphene materials, researchers have developed a variety of modification strategies. Compared with pure graphene, Wu et al. [22] reported the development of a PVP-GR composite material that utilizes the synergistic interaction between graphene and polyvinylpyrrolidone (PVP) to achieve superior dispersion and film-forming capabilities. By employing a straightforward drop-casting strategy, this composite forms a uniform and stable film on the surface of a glassy carbon electrode (GCE) (Figure 4). The PVP-GR/GCE-modified electrode demonstrated outstanding catalytic activity for the electrochemical oxidation of AA, DA, and UA, enabling the simultaneous and precise detection of these three biomolecules. Nonetheless, drop-cast coatings can be sensitive to film thickness uniformity and drying conditions, which may lead to batch-to-batch variability and potential diffusion barriers; therefore, standardized fabrication protocols and reproducibility evaluation across independent batches are important for practical use. Hsieh et al. [23] compare the sensing performance of graphene, multiwalled carbon nanotubes (MWCNTs), and graphene−multiwalled carbon nanotube (GR-MWCNT) composite modified glassy carbon electrodes for the simultaneous electrochemical determination of AA, DA and UA, aiming to mitigate signal overlap on unmodified electrodes. Using differential pulse voltammetry, the GR-MWCNT/GCE provided the best analytical performance, offering clearly separated oxidation peaks at low potentials alongside improved sensitivity and detection capability. The sensor also exhibited good stability and anti-interference performance and was demonstrated in diluted real serum and urine matrices, although broader validation across more diverse clinical samples and long-term use conditions would further strengthen its translational relevance. Nonetheless, drop-cast coatings are often sensitive to film uniformity and drying conditions, which can cause batch-to-batch variability and mass-transport limitations; thus, standardized fabrication and inter-batch reproducibility assessments are essential for practical deployment.

In addition, three-dimensional graphene-based materials, such as graphene hydrogels (GH) and graphene aerogels (GA), exhibit tremendous potential in applications like catalysis [95], batteries [96], electrochemical capacitors [97] and sensors [98]. This unique structure not only provides more active sites, but also significantly promotes material transfer [99]. Feng et al. [24] developed a porous nitrogen-doped graphene aerogel (HNGA), whose unique hierarchical porous structure and composition make it an ideal material for electroanalytical applications. The HNGA-modified glassy carbon electrode (HNGA/GCE) exhibited significantly enhanced electrochemical responses for AA, DA, and UA, enabling the simultaneous detection of these three biomolecules. Furthermore, their oxidation peaks were completely separated, with distinct peak potential differences. These studies provide new insights and approaches for the development of high-performance biosensors. Notably, porous 3D frameworks can face mechanical/structural stability and nonspecific adsorption issues in biofluids, making antifouling design and long-term operational validation essential for practical deployment.

**Table 5:** Performance comparison for UA detection with different sensors.

Sensing Materials	Methods	Linear Range (µM)	LOD (µM)	Ref.
CuO/GCE	CV	1–35,100	0.6	[19]
UOx/Fc/Cu_2_O/GCE	DPV	10–1000	0.0596	[20]
GNSs/CC	DPV	20–1000 0.5–20; 0.5–20	0.31 (AA) 0.01 (DA) 0.03 (UA)	[21]
PVP-GR/GCE	LSV	4.0–1000 0.02–0.2; 0.2–100 0.04–1.0; 1.0–100	0.8 (AA) 0.002 (DA) 0.02 (UA)	[22]
GR-MWCNT/GCE	DPV	100–1000 5–50 50–500	6.71 (AA) 0.58 (DA) 7.30 (UA)	[23]
HNGA/GCE	DPV	50–1500 5–50 5–50	16.7 (AA) 0.22 (DA) 0.12 (UA)	[24]

### 3.4. Hydrogen Peroxide (H_2_O_2_)

Hydrogen peroxide (H_2_O_2_), as a reactive oxygen species (ROS), is widely present in biological platforms and plays a pivotal role in various physiological processes such as cell signaling, growth regulation, immune activation, and apoptosis [100,101,102,103]. However, abnormally elevated H_2_O_2_ levels can harm the body, causing cellular damage [104], inflammatory diseases [105] and cancer [106]. As an effective biomarker for the early diagnosis and treatment of cancer, monitoring H_2_O_2_ concentration is of great significance for clinical diagnostics and cancer therapy. Compared to existing analytical strategies, electrochemical sensing technology offers high sensitivity, excellent selectivity, simplicity, and cost-effectiveness, making it a promising approach for dynamic H_2_O_2_ concentration analysis.

**Figure 5:**
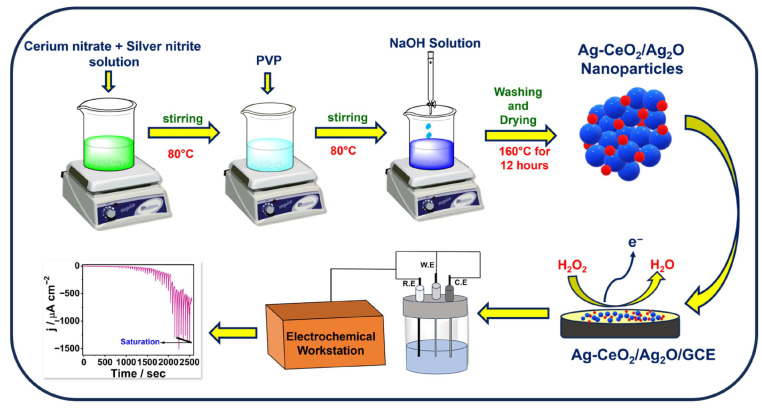
Electrocatalytic mechanism of the Ag–CeO_2_/Ag2O electrode for H_2_O_2_ detection. Reprinted with permission from Ref. [29]. Copyright © 2025 by the authors.

In recent years, various metal materials (e.g., Au, Pd, and Pt) or metal oxides (e.g., Cu_2_O and MnO_2_) with outstanding catalytic activity have been utilized in electrochemical sensors to enhance H_2_O_2_ detection [107,108,109]. In terms of material design and preparation, researchers have proposed several innovative strategies. Liang et al. [25] developed a rapid and simple one-step electrochemical deposition strategy to successfully prepare a Cu_2_O/AuCu/Cu composite material-modified glassy carbon electrode for H_2_O_2_ detection. The layered structure of this material not only ex-poses abundant active sites but also takes full advantage of the excellent conductivity of Au and Cu, significantly promoting the catalytic reaction and electron transfer process of H_2_O_2_, thereby enhancing the detection efficiency. Nevertheless, electrodeposited mul-tilayer composites can be sensitive to deposition parameters (potential, time, electro-lyte), which may cause batch-to-batch variability, and their long-term adhe-sion/stability under continuous operation or in protein-rich matrices warrants further validation. Wu et al. [26] employed a template-in situ modification strategy to synthesize Pt/MoSe_2_ nanowires, applying them for the first time in H_2_O_2_ detection (Figure 5). In this hybrid system, the incorporation of Pt nanoparticles markedly enhanced the electrical conductivity and catalytic activity, while the MoSe_2_ framework provided a highly porous architecture with abundant exposed active sites. This synergistic integration endowed the Pt/MoSe_2_ nanowires with excellent selectivity and anti-interference capability toward H_2_O_2_ detection, demonstrating their strong potential for practical biosensing applications. Nevertheless, considering the involvement of Pt and the potential sensitivity of MoSe_2_-based frameworks to operating environments, additional validation of long-term stability and reproducibility in complex matrices would further strengthen its practical relevance.

To further enhance detection performance metrics, Xie et al. designed a novel electrochemical sensor based on cobalt metal–organic frameworks (Co-MOF) supported by bimetallic Au_3_Pt_7_ nanoparticles (NPs). Studies revealed that the bimetallic Au_3_Pt_7_ NPs demonstrated higher electrocatalytic activity than single-metal materials. The constructed Au_3_Pt_7_/Co-MOFs/GCE sensor successfully realized real-time monitoring of H_2_O_2_ concentration and exhibited ideal properties in cancer sample detection [27]. Notably, MOF-supported catalytic interfaces can be sensitive to framework integrity and interfacial mass transport under prolonged electrochemical operation, and the claim of “real-time” monitoring would be further strengthened by extended continuous measurements, antifouling assessments, and benchmarking against established reference methods in relevant biological matrices. More recently, Zhang et al. [28] introduced a fast and environmentally friendly strategy for H_2_O_2_ detection based on AgNPs/RGO biosensor. This strategy synthesized AgNPs on graphene oxide (GO) through a hydrothermal process, then electroplated and converted them into AgNPs/rGO and modified them on the electrode surface. This simple and environmentally friendly preparation strategy not only provides a new approach for H_2_O_2_ detection but also lays the foundation for developing multi-target detection sensors. Manibalan et al. [29] developed a non-enzymatic Ag-doped CeO_2_/Ag_2_O nanocomposite-modified glassy carbon electrode (Ag-CeO_2_/Ag_2_O/GCE) and systematically evaluated its sensing performance using cyclic voltammetry and amperometry. The enhanced electrocatalytic response was attributed to an increased density of accessible active sites and accelerated electron transfer. The platform also exhibited high selectivity against common coexisting electroactive species, together with good repeatability and storage stability, supporting its applicability for practical analysis. Notably, although these results are encouraging, the real-sample validation was mainly carried out in tomato extract spiked with a commercial antiseptic liquid, and further verification in clinically relevant biofluids under prolonged operation would strengthen its translational significance.

In parallel, framework-assisted immobilization and conductive-network integration provide another effective route to improve interfacial robustness. A representative example is the COF-based nanoflower composite COFTZT-DVA/CNT@PB, in which the nanoflower-like COF offers a high surface area for analyte enrichment and structural hosting, CNTs accelerate charge transport, and Prussian blue nanoparticles are more stably retained on the electrode surface than with PB alone [30]. Electrochemical characterization and parameter optimization were performed using CV and chronoamperometry, enabling sensitive H_2_O_2_ detection with good reproducibility and anti-interference capability. Nevertheless, because the current validation was largely conducted in buffered conditions, systematic antifouling evaluation and performance benchmarking in untreated or lightly treated real biofluids would further strengthen the case for practical biosensing deployment.

These research advancements have provided new material platforms and methodological support for high-performance H_2_O_2_ detection, promoting the application and development of electrochemical sensing technology in the biomedical field (Table 6). Nevertheless, to enable reliable biomedical translation, future studies should place greater emphasis on long-term operational stability, antifouling robustness in complex biofluids, and rigorous inter-device reproducibility and reference-method benchmarking.

**Table 6:** Performance comparison for H_2_O_2_ detection with different sensors.

Sensing Materials	Methods	Linear Range (µM)	LOD (µM)	Ref.
Cu_2_O/AuCu/Cu	I-t	0.3-10	0.14	[25]
Pt/MoSe_2_	I-t	8–6818	2.56	[26]
Au_3_Pt_7_/Co-MOFs/GCE	I-t	0.1–5000 5000–60,000	0.02	[27]
AgNPs/rGO/GCE	CV	1–276	0.18	[28]
Ag-CeO_2_/Ag_2_O/GCE	CV, I-t	0.01–500	6.34	[29]
COFTZT-DVA/CNT@PB/GCE	I-t	2.38–1050	0.79	[30]

### 3.5. Lactic Acid (LA)

Lactic acid, an important biomolecule prevalent in the human body [110], exhibits concentration levels that are closely linked to various physiological and pathological processes. In clinical medicine, abnormal lactic acid levels are significantly associated with the onset and progression of conditions such as heart failure, liver diseases [111], sepsis [112], and tissue hypoxia [113]. Furthermore, as a key metabolite in anaerobic metabolism, when the energy demands of tissues surpass the supply capacity of aerobic respiration, there is a marked increase in lactic acid concentration. Excessive accumulation of lactic acid can lead to lactic acidosis [114]. Consequently, rigorous monitoring of lactic acid concentrations holds substantial clinical significance within the biomedical field. Additionally, in the food and fermentation industries, lactic acid serves as a specific indicator for bacterial fermentation processes. It is used to evaluate the freshness and quality of various products, including fermented dairy products, alcoholic beverages, pickled meats, vegetables, and more [115]. Given its critical role across multiple domains, developing efficient and reliable strategies for detecting lactic acid has emerged as a prominent research focus.

Traditional electrochemical sensors for lactic acid primarily rely on immobilized enzymes combined with reactants, catalyzing enzymatic reactions to produce a selective current proportional to the lactic acid concentration [116]. However, the instability and high cost of lactic acid enzymes limit the widespread application of these biosensors [117,118]. In contrast, enzyme-free sensors have garnered significant attention due to their low manufacturing cost, strong electrochemical redox performance, durability, and excellent reproducibility [119,120,121]. With advancements in nanotechnology, non-enzymatic electrochemical sensors utilizing nanomaterials have made significant strides in enhancing detection capabilities for lactic acid. In the design of nanomaterials, researchers have developed a variety of high-performance sensing platforms. Xiao et al. [31] successfully constructed a sensing platform using gold-platinum nanoparticles modified molybdenum disulfide nanosheets (MoS_2_-AuPt). The AuPt nanoparticles not only significantly enhanced electron transfer rates but also enabled the non-enzymatic electrochemical oxidation of lactic acid, providing an efficient electrocatalyst for lactic acid detection. This sensor demonstrated outstanding analytical performance metrics, with a detection limit as low as 0.00033 mM and a rapid response time (<15 s). This strategy provides a new technical path for future integration into wearable devices and microneedle electrochemical sensors. Nevertheless, noble-metal-based catalysts can increase cost and may suffer from surface poisoning or performance drift in complex biofluids; therefore, long-term stability and antifouling validation under realistic sweat/serum conditions are needed to substantiate practical wearability claims.

To further enhance sensing performance metrics, Tao et al. synthesized a novel copper and cobalt oxide-doped multidimensional multi-walled carbon nanotube nanocomposite (Co_3_O_4_/CuO@ MWCNTs NCs). This lightweight, highly sensitive nanomaterial exhibited excellent electrocatalytic activity for lactate oxidation and was successfully applied for the first time to the detection of L-lactate in simulated human sweat. The preparation strategy is characterized by mild reaction conditions and simplicity, laying a foundation for large-scale production of high-performance sensors [32]. However, tests in simulated sweat cannot fully capture variability in real perspiration (pH, salts, proteins, metabolites) and mechanical deformation during wear; thus, demonstration in human sweat with inter-individual variability and on-body motion stability would strengthen translational relevance. In terms of sensor construction strategies, the optimization of manufacturing process and the fixation of biometric components are the keys to ensure the repeatability of performance. Faisal et al. innovatively combined platinum nanoparticles with a chitosan/ZnTiO_3_ nanocomposite (NCs) to develop an L-lactate electrochemical sensor based on Pt@Chitosan/ZnTiO_3_ NCs/GCE. By introducing a conductive PEDOT: PSS adhesive coating, the sensor achieved efficient detection of L-lactate within a range of 0.3–2.4 mM, providing a reliable tool for clinical diagnostics [33]. Recently, paper-based electrochemical biosensors have gained attention due to their abundant materials and low costs. Jia et al. integrated AuNP/Cu-TCPP(Fe) hybrid nanoparticles with a paper-based electrochemical analytical device (ePAD) to develop a novel sensor for non-invasive detection of lactate in sweat. This sensor demonstrated a wide linear detection range (0.013 nM–100 mM) and an ultra-low detection limit of 0.9 pM, making it one of the most advanced lactate detection strategies reported to date [34].

**Figure 6:**
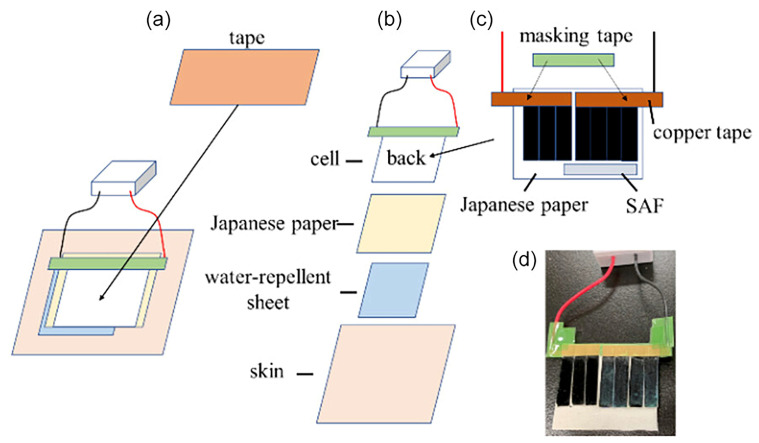
Self-powered lactate sensor for on-body tests. (**a**) Sensor device on skin. (**b**) Sensor device layers. (**c**) Front view of the sensing part. (**d**) Photo of the assembled self-powered sensor. Reprinted ith permission from Ref. [122]. Copyright © 2025 by the authors.

In addition, wearable sensors are widely used for real-time monitoring of lactate in sweat in healthcare and sports physiology. Wu et al. designed a flexible wearable LOx@CS PC sensor composed of a PC nanoporous electrode and an LOx@CS hydrogel. This sensor is capable of simultaneously detecting sweat lactate and body temperature, offering long-term durability (13 days), excellent selectivity, a broad linear range (0.01–35 mM), and a low detection limit (0.144 µM) [35]. Beyond conventional battery-powered designs, Shitanda [122] and co-workers reported a wearable self-powered lactate biosensor for continuous sweat monitoring, built on a paper-based biofuel cell (BFC) and integrated with a voltage booster and a Bluetooth transmitter to enable battery-free wireless readout to a smartphone (Figure 6). By improving the bioanode stability via mediator/crosslinking–trapping strategies, the BFC delivered enhanced output and an on-body exercise test revealed a correlation between sweat and blood lactate trends. Notably, the sensor response remained pH-dependent and required manual pH-based calibration selection/adjustment, indicating that integrated pH compensation and extended antifouling/long-duration on-body validation would further strengthen translational relevance.

Overall, these advances provide diversified material platforms and device formats for lactate sensing and continue to drive the development of electrochemical technologies for lactate analysis (Table 7). Nevertheless, translating high performance from proof-of-concept studies to real-world deployment remains nontrivial. Because many studies still rely on simulated or spiked samples, there is an urgent need for larger-cohort, long-term evaluations of stability and repeatability in real wearing scenarios, together with benchmarking against standard reference methods to substantiate clinical and exercise-monitoring utility.

**Table 7:** Performance comparison for LA detection with different sensors.

Sensing Materials	Methods	Linear Range (µM)	LOD (µM)	Ref.
MoS_2_-AuPt	SWV	5–3000	0.33	[31]
Co_3_O_4_/CuO@MWCNTs NCs	CV	0.001–100,000	0.000055	[32]
Pt@ Chitosan/ZnTiO_3_NCs/GCE	DPV	300–12,000	22.36	[33]
Cu-TCPP(Fe)/Au/LOx	CV	0.000013–100,000	0.00000091	[34]
Lox @ CS PC	CV	10–35,000	0.144	[35]

### 3.6. Cholesterol

Cholesterol, as an essential lipid component of animal and human cell membranes, serves as a critical biomarker for various diseases. Studies have exhibited that excessive cholesterol levels in the bloodstream significantly increase the risk of arterial diseases [123], making it a major contributing factor to coronary heart disease, hypertension, atherosclerosis, and lipid metabolism disorders [124,125]. Therefore, developing accurate and rapid cholesterol detection strategies holds great clinical significance. Currently, the quantitative detection of cholesterol primarily relies on biosensors based on cholesterol oxidase (ChOx) or cholesterol esterase (ChEt). These enzymatic electrochemical devices function by immobilizing enzymes on modified electrodes, which catalyze trace cholesterol in the solution and convert it into measurable electrochemical signals [126].

In recent years, nanostructured interfaces have been widely adopted to improve enzyme loading, interfacial electron transfer, and overall signal transduction, thereby enhancing the sensitivity and stability of enzymatic cholesterol sensors. For example, Hang et al. [36] reported a high-sensitivity cholesterol biosensor on disposable screen-printed carbon electrodes (SPCEs), where Au-Ag@Au core–shell nanoparticles and a Prussian blue/PEDOT (PB-PEDOT) nanocomposite layer were integrated to boost conductivity and interfacial charge transfer, while chitosan was used to immobilize cholesterol oxidase (ChOx), enabling both amperometric and impedimetric readouts. Importantly, the platform was validated using paired human serum and saliva samples and the reported serum–saliva correlation provides supportive evidence for noninvasive cholesterol monitoring. Beyond disposable architectures, enzyme–nanomaterial synergy has been exploited to increase effective enzyme loading and accelerate interfacial kinetics. Ayyandurai et al. [37] constructed a CoFe_2_O_4_@MoS_2_/Au/ChOx-modified GCE, where the high surface area and enhanced conductivity of the hybrid nanocomposite helped improve biosensor performance, and cholesterol quantification was achieved using DPV. The authors systematically evaluated common interferents and reported encouraging storage stability and reproducibility across multiple independently prepared sensors, supporting the robustness of the interface engineering approach. Nevertheless, broader validation in clinically relevant matrices and under continuous operation conditions would further strengthen the evidence for real-world deployment beyond controlled buffer-based measurements.

**Figure 7:**
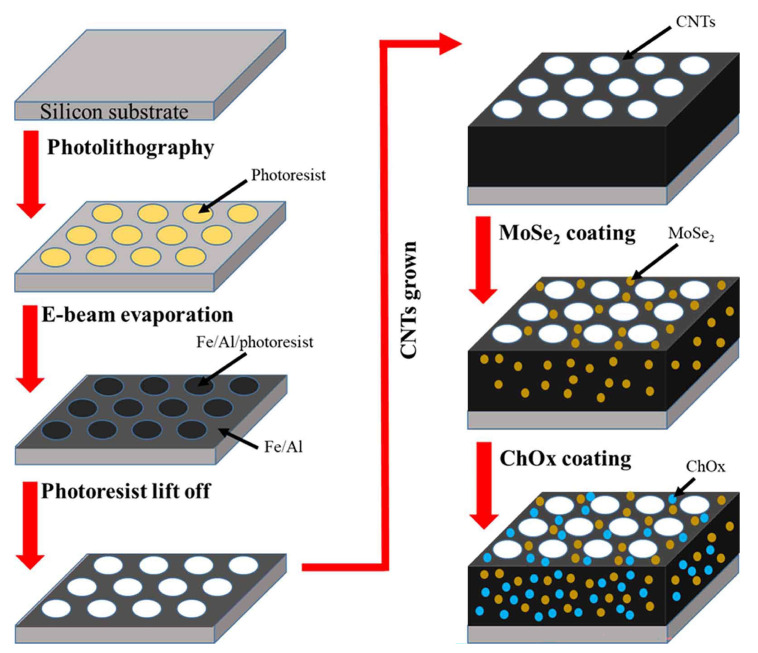
The schematic diagram of the electrode fabrication process. Reprinted with permission from Ref. [38]. Copyright © 2024 by the authors.

At the micro-/nanostructure level, Hu et al. [38] employed photolithography-defined hive-shaped vertically aligned CNT arrays followed by CVD of MoSe_2_ and ChOx immobilization to create a cholesterol biosensor, and correlated the CV peak current response with cholesterol concentration (Figure 7). The authors attributed the performance gains to the enlarged surface area of the hive architecture and the pseudocapacitive contribution of MoSe_2_, achieving sensitive detection across both low and human-relevant concentration windows. Still, this platform involves multi-step microfabrication and cholesterol solubilization using surfactant/alcohol-assisted preparation, and the enzyme immobilization step requires extended drying, which may affect scalability and matrix comparability; therefore, further demonstration in real biofluids and manufacturability-oriented optimization would be valuable. These studies not only highlight the pivotal role of the synergy between nanomaterials and enzymes in sensor design but also provide new insights into the development of high-performance cholesterol detection platforms.

Although enzymatic analysis strategies exhibit high sensitivity and selectivity, the inherent fragility and degradability of enzymes result in poor long-term stability, limiting their widespread application in biosensing. To address this issue, researchers have explored non-enzymatic cholesterol biosensors as an alternative, which primarily rely on the catalytic performance metrics of modified composite materials such as metal oxides, carbon materials, and polymers, effectively circumventing the instability of enzymes. In the development of non-enzymatic sensors, the synergistic effects of nanomaterials have emerged as a key factor in enhancing performance metrics. Shahriarinour et al. developed a novel electrochemical sensor by modifying pencil graphite electrodes (PGE) with copper nanoparticles (CuNPs) and indole (IND). This sensor leveraged the synergy between IND and CuNPs to enable the direct quantitative detection of cholesterol in real samples [39]. In another study, Shiri et al. developed a novel electrochemical response used a metal oxide composite. They prepared NiO/CuO nanocomposites passed electrospinning and leveraged the synergistic effect between NiO and CuO to enhance the oxidation current. This significantly improved the electrocatalytic activity for cholesterol oxidation, enabling efficient cholesterol detection [40]. However, metal-oxide catalytic interfaces may undergo surface passivation, structural evolution, or ion leaching during prolonged electrochemical operation, which can compromise long-term reproducibility; thus, extended stability testing and inter-batch reproducibility assessment would further strengthen practical applicability. In recent studies, significant progress has also been made in non-enzymatic sensors based on biorecognition elements, Bernardo et al. [41] developed a novel sensor based on cholesterol-recognition peptides, utilizing screen-printed carbon electrodes (SPEs) modified with porous poly (L-lactic acid) (PLLA) nanomembranes as peptide immobilization carriers. Cholesterol detection was achieved through the specific binding between the peptide and cholesterol. This innovative approach provides new insights into cholesterol sensing and highlights the potential of biorecognition elements in non-enzymatic sensors.

These studies indicate that non-enzymatic cholesterol sensors hold promise in overcoming the instability of enzymatic sensors, offering solutions for various application scenarios (Table 8). Future developments should place greater emphasis on rational design, including the thoughtful design of nanomaterial composites and the incorporation of biorecognition elements, to develop next-generation cholesterol detection technologies with high sensitivity, high selectivity, exceptional stability, and favorable cost-effectiveness.

**Table 8:** Performance comparison for Cholesterol detection with different sensors.

Sensing Materials	Methods	Linear Range (µM)	LOD (µM)	Ref.
ChOx-Chit/PB-PEDOT/Au-Ag@Au NPs/SPCE	I-t	10–1000	3.3	[36]
CoFe_2_O_4_@MoS_2_/Au-ChOx	DPV	5–100	0.09	[37]
ChOx/MoSe_2_/CNTs	CV	0–100	0.082	[38]
PIND/CuNPs/PGE	CV, SWV	0.015–0.195	0.00498	[39]
NiO/CuO/GCE	CV	800–65,000	5.9	[40]
C-pept/PLA NM/SPE	EIS	2–6	6.31	[41]

### 3.7. Glutathione (GSH)

Glutathione (GSH) is a tripeptide composed of glutamic acid, cysteine, and glycine connected by γ-amide bonds. The thiol group (-SH) in its molecular structure imparts unique biological activity, allowing GSH to play a crucial role in various physiological processes, including antioxidant defense, detoxification, and regulation of cellular redox status [127,128]. GSH is widely present in human cells and plays a crucial role in maintaining protein structure, stabilizing the redox state, and regulating immune function, among other metabolic processes [129]. Research has exhibited that low levels of intracellular GSH can lead to liver damage, diabetes, Parkinson’s disease and even cancer [130]. Given the significant application value of GSH in the pharmaceutical field, it is not only used in medicine but also serves as a fundamental ingredient in functional foods, widely applied in anti-aging, immune enhancement, and anti-tumor therapies [131]. Moreover, many vegetables and fruits contain substantial amounts of GSH, and dietary intake of GSH may be an economical and effective way to maintain its balance and protect the body from GSH deficiency. Adequate supplementation of GSH in the diet is crucial for maintaining human health. Therefore, developing highly sensitive and selective analytical strategies to detect GSH in various biological media or food samples holds important research value in clinical diagnostics and food screening.

In recent years, significant progress has been made in the development of electrochemical sensing platforms for glutathione based on various material platforms. Rajendran et al. developed an electrochemical biosensor platform by modifying a glassy carbon electrode with nanoporous PEDOT through electrochemical deposition, followed by the self-assembly of gold nanoparticles (AuNPs) into the nanoporous PEDOT structure. This AuNP-PEDOT/GCE platform was successfully applied to GSH detection [42]. Additionally, Li et al. built a novel electrochemical sensor based on silver metal–organic frameworks (Ag-MOF) by exploiting the stronger affinity between Ag^+^ and GSH compared to that between Ag^+^ and Cl^−^. The interaction between the Ag-GSH complex and Ag-H_3_BTC induced the collapse of the Ag-MOF structure, leading to significant current changes. This specificity and competitive reaction, along with the collapse effect, enhanced the sensitivity and selectivity of the sensor for GSH detection [43]. Notably, because this sensing mechanism is governed by coordination competition in electrolyte environments, further evaluation against representative biothiols and under physiologically relevant ionic/protein backgrounds would help clarify analytical specificity in real matrices. However, the irregular crystal structure and poor conductivity of metal–organic frameworks limit their application in electrochemical sensors. To address this issue, studies have demonstrated that adding polyvinylpyrrolidone (PVP) during the synthesis of MOFs can promote morphological control and enhance the material’s conductivity. Based on this finding, Huang et al. [44] synthesized a PVP-doped copper-based MOF (N-Cu-MOF), which was pyrolyzed to form CuNPs@NPC. The reaction between CuCl and GSH produces a non-electroactive Cu-GSH complex, resulting in a decrease in the Cu peak current, while the peak current of the internal reference APAP remains stable. The ΔICu/IAPAP ratio is used as the signal, enabling ultra-sensitive detection of GSH. Although the proportional reading method can effectively suppress the baseline drift, the introduction of internal reference also brings new challenges, which increases the operational flux and complexity of sample processing, and increases the efficiency of sample processing, the signal itself may also be disturbed by coexisting electroactive substances. Therefore, standardized method validation in a variety of representative samples is essential.

In a recent study, Mahmoud et al. proposed a novel anti-fouling, porous, and conductive nanocomposite material for the determination of GSH. The sensor, composed of chitosan (Chit), polyethylene glycol (PEG-OH), porous magnesium metasilicate (PMMS) loaded with Cu(II), and silver nanoparticles (AgNPs), was successfully applied to measure GSH in dietary supplements and human serum samples [45]. However, multi-component composites can face batch-to-batch variability and potential metal-ion leaching, highlighting the need for extended durability testing and reference-method benchmarking.

**Figure 8:**
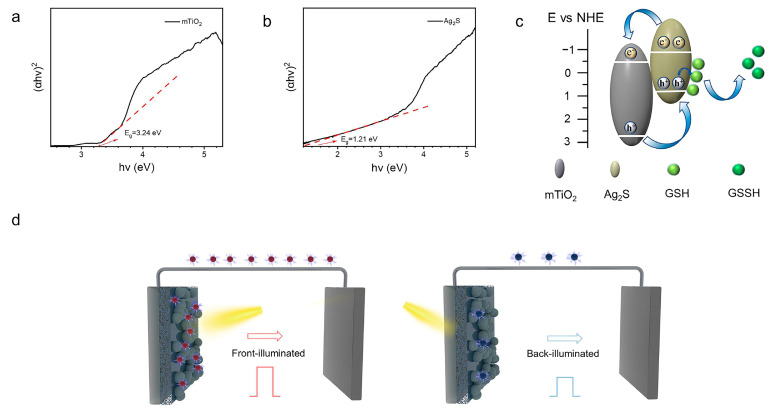
Band gap values of (**a**) mTiO_2_ and (**b**) Ag_2_S. (**c**) The mechanism illustration of the mTiO_2_/Ag_2_S PEC sensor. (**d**) The ratiometric PEC sensing mechanism. Reprinted with permission from Ref. [46]. Copyright © 2024 by the authors.

Beyond conventional single-signal electrochemical readouts, Huang et al. [46] developed a new ratiometric PEC sensor based on front and back lighting to regulate carrier transport for glutathione detection (Figure 8). The sensor enhances the photocurrent signal through the mTiO_2_/Ag_2_S heterojunction, improves the selectivity by the specific interaction between Ag_2_S and sulfhydryl, and realizes the self-calibration detection by the ratio of front and back illumination photocurrent, which effectively reduces the interference, this strategy provides a new idea for the design of ratiometric sensing platform. To achieve accurate clinical detection of glutathione, Farooq et al. [47] designed a GO-SiO_2_@AgNPs modified carbon fiber ultramicroelectrode. By virtue of the synergistic effect of the three components, the electrode surface area and electron transfer efficiency are improved, enhancing the detection sensitivity. The sensor has a detection limit of 0.17 μM for GSH and a linear range of 0.25–3 µM, which can be successfully applied to the detection of human serum samples and real-time monitoring of GSH in mice, providing a reliable tool for the clinical diagnosis of oxidative stress-related diseases.

These research advancements provide new ideas and strategies for the development of GSH detection technologies, advancing the progress of electrochemical sensing for glutathione (Table 9). Future work should establish a standardized assessment process, systematically carry out anti-fouling and long-term stability research, and strictly verify the standard method in the actual sample system. With continued optimization of material platforms and sensing mechanisms, it is expected that more efficient, sensitive, and stable electrochemical GSH sensors will be developed in the future, offering strong technical support for clinical diagnostics and food screening.

**Table 9:** Performance comparison for GSH detection with different sensors.

Sensing Materials	Methods	Linear Range (µM)	LOD (µM)	Ref.
AuNP-PEDOT/GCE	I-t	0.5–10 3000–15,000	0.173	[42]
Ag-MOF	DPV	0.0001–1	0.000018	[43]
CuNPs@ NPC/GCE	DPV	0.0001–10	0.000067	[44]
Cu(II)-PMMS/AgNPs/PEG-OH/Chit	SWV	0.1–125	0.03	[45]
mTiO_2_/Ag_2_S	PEC	10–10,000	6.39	[46]
GO-SiO_2_@AgNPs	I-t	0.25–3	0.17	[47]

## 4. Conclusions

In recent years, electrochemical strategies for biomolecule detection have progressed markedly, driven by advances in electrode/material engineering, nanostructure-enabled electrocatalysis, and signal-amplification schemes. These developments have strengthened the competitiveness of electrochemical sensing in terms of sensitivity, cost-effectiveness, and scalability, thereby expanding its utility across clinical diagnostics, food safety, and environmental monitoring. Despite the considerable promise of electrochemical sensing, several major challenges remain. First, matrix effects in complex biofluids (e.g., blood, serum, and saliva) represent a critical bottleneck. Non-target constituents can readily interfere with molecular recognition and signal transduction at the sensor interface, highlighting the need for further efforts to achieve highly specific detection under strong background interference. Second, the long-term stability and batch-to-batch reproducibility of sensors require substantial improvement, particularly when performance is evaluated using real samples, where fluctuations in environmental conditions (e.g., pH and temperature) and interfacial fouling can lead to signal drift and performance degradation. In addition, the development of multifunctional integrated sensors is still at an early stage, which limits their applicability in complex real-world scenarios.

To address these challenges, future research should focus on the following concrete directions. First, electrode interfacial engineering aimed at enhancing anti-interference capability and analytical specificity is essential. This includes developing advanced surface-modification strategies and next-generation antifouling materials to suppress nonspecific adsorption of matrix constituents, thereby improving sensor robustness in complex biological samples. Second, simultaneous multi-analyte detection should be advanced by constructing electrochemical sensor arrays based on multichannel electrode chips or multi-signal transduction schemes, in which each channel or signal is dedicated to a specific target, enabling high-throughput parallel detection of multiple biomolecules. In addition, continued efforts are needed to promote the miniaturization and smart integration of point-of-care (POC) devices. Integrating electrochemical sensors with microfluidic chips can enable automated sample pretreatment and reduce operational complexity; furthermore, coupling portable electrochemical workstations with wireless communication modules and data-analysis algorithms may facilitate smart POC systems capable of real-time quantification and data transmission, thereby supporting deployment in resource-limited settings. Improving long-term stability is also of paramount importance. Robust substrate materials and reliable encapsulation/packaging technologies should be explored to mitigate electrode corrosion and interfacial degradation, enhancing both storage stability and operational lifetime; meanwhile, standardized fabrication protocols for scalable manufacturing are necessary to ensure batch-to-batch reproducibility. Finally, deeper interdisciplinary convergence across materials science, nanotechnology, biotechnology, and clinical medicine will be pivotal for enabling real-time, dynamic biomolecular monitoring and for delivering new tools that support precision medicine and fundamental biomedical research. Addressing these challenges will remain a central focus of future research in this field.

In conclusion, with the in-depth interdisciplinary integration of materials science, nanotechnology, biotechnology, and biomedicine, electrochemical approaches for biomolecule detection will play an increasingly pivotal role in future precision disease diagnosis and treatment, basic biomedical research, and nanomedical translation, providing strong technical support for human health protection and the advancement of life sciences.

## Figures and Tables

**Table 2:** Comparison of common electrochemical techniques.

Technique	Sensitivity	Antiinterference	Application Scenarios	Limitations
CV	Moderate	Low	Reaction mechanism study	Peak overlap in complex systems
EIS	High	Moderate	Interface performance analysis	Complex data interpretation
DPV	Very high	High	Trace small-molecule quantification	Relatively long measurement time

## Data Availability

No new data were created or analyzed in this study. Data sharing is not applicable to this article.

## Abbreviations

CV	Cyclic voltammetry
DPV	Differential pulse voltammetry
EIS	Electrochemical impedance spectroscopy
CE	counter electrode
WE	working electrode
RE	reference electrode
3E	three-electrode
AC	alternating current
POCT	point-of-care testing
SNR	signal-to-noise ratio
MOFs	Metal–organic frameworks
CNTs	Carbon nanotubes TOM
TMOs	transition metal oxides
DA	Dopamine
HPLC	high-performance liquid chromatography
p-L-Trp	polymerized L-tryptophan
OECT	organic electrochemical transistor
CSF	carbonized silk fabric
RGO	reduced graphene oxide
GOx	glucose oxidase
FAD	flavin adenine dinucleotide
LOD	limit of detection
AuNPs	gold nanoparticles
AMWCNTs	amine-functionalized multi-walled carbon nanotubes
SPE	screen-printed electrodes
XSBR	carboxylated butadiene styrene rubber
UA	Uric acid
AA	ascorbic acid
GN	Graphene
CC	carbon cloth
3D	three-dimensional
GNSs	graphene nanosheets
CVD	chemical vapor deposition
PVP	polyvinylpyrrolidone
GCE	glassy carbon electrode
MWCNYs	multiwalled carbon nanotubes
GH	graphene hydrogels
GA	graphene aerogels
HNGA	porous nitrogen-doped graphene aerogel
H_2_O_2_	Hydrogen peroxide
ROS	reactive oxygen species
Co-MOF	cobalt metal–organic frameworks
NPs	nanoparticles
GO	graphene oxide
NCs	nanocomposite
ePAD	paper-based electrochemical analytical device
BFC	biofuel cell
ChOx	cholesterol oxidase
ChEt	cholesterol esterase
SPCEs	screen-printed carbon electrodes
MoS_2_	molybdenum disulfide
PGE	pencil graphite electrodes
CuNPs	copper nanoparticles
IND	indole
PLLA	porous poly (L-lactic acid)
GSH	Glutathione
Ag-MOF	silver metal–organic frameworks
Chit	chitosan
PEG-OH	polyethylene glycol
PMMS	porous magnesium metasilicate
POC	point-of-care
